# Immunostaining and time-lapse analysis of vinblastine-induced paracrystal formation in human A549 cells

**DOI:** 10.3892/ol.2014.2549

**Published:** 2014-09-18

**Authors:** YUKA NAKAMURA, YASUHITO ISHIGAKI

**Affiliations:** Medical Research Institute, Kanazawa Medical University, Ishikawa 920-0293, Japan

**Keywords:** vinblastin, tubulin, paracrystal, time-lapse microscopy, A549 cells

## Abstract

Vinblastine is a vinca alkaloid that binds to tubulin and inhibits microtubule formation in cells. Vinblastine treatment results in the formation of paracrystalline aggregates in the cells, which are formed from tightly packed tubulin molecules. Mitotic spindle assemblies in treated cells are disrupted and cell cycle progression is arrested at the mitosis phase. Vinblastine is therefore widely used for cancer treatment. However, the mechanism underlying paracrystal formation has not been fully elucidated. The present study attempted to observe paracrystal formation in human A549 cells. Initally, paracrystal formation was detected using the anti-tubulin antibody. Secondly, the exogenousuly expressed RFP-conjugated tubulin also formed paracrystals. Additionally, immunostaining with the anti-RBM8A antibody overlapped with paracrystal images obtained from RFP conjugated tubulin. This suggested that the localization of the RBM8A proteins was adjacent to the tubulin molecules prior to vinblastine treatment. Furthermore, a time-lapse analysis was developed for paracrystal formation in viable human A549 cells. This was achieved using exogenous expression of fluorescent proteins conjugated with tubulin and time-lapse microscopy. It may be concluded that the indicated method was successful for the real-time analysis of paracrystal formation in human cells.

## Introduction

The vinca alkaloid, vinblastine, is a chemical analogue of vincristine. Vinblastine binds to tubulin, which inhibits microtubule assembly. Vinblastine is an anti-tumor drug that is widely used in cancer chemotherapy, as it inhibits cell proliferation via a mitotic block (1,2). This anti-microtubule agent is used to treat various types of cancer, including Hodgkin’s lymphoma, Kaposi sarcoma, breast cancer, head and neck cancer, Langerhans cell histiocytosis and testicular cancer (3–6). In addition, it is used to treat non-small cell lung cancer (7).

Although the mechanism behind it is not entirely understood, one characteristic of vinblastine treatment is the formation of giant paracrystalline aggregates in the cell cytoplasm. These paracrystals consist of tubulin molecules and can be detected by tubulin immunostaining under a light microscope or by transmission electron microscopy (8–13). The mechanism of paracrystal formation has been ascribed to the capacity of vinblastine to depolymerize mitotic spindle microtubules. To understand the mechanism of paracrystalline aggregate formation in more detail, an experimental system for observing their formation is required.

RBM8A, a member of the RNA binding motif (RBM) family (14), localizes to mRNA through its RNA binding domain (15). RBM8A binds to spliced mRNA and maintains this binding until the bound mRNA is degraded (16). It was previously demonstrated that depleting RBM8A proteins in *Drosophila* SL2 cells resulted in impaired cell growth (17). Furthermore, Sudo *et al* performed loss-of-function screening for genes involved in apoptosis and growth for a human mesothelioma cell line (18). In addition to the *COPA* gene, *RBM8A* was also shown to contribute to cell growth, as observed by gene silencing experiments using RNAi. In addition, these results were confirmed using human tumor cell lines and the contribution of G_2_/M phase progression was indicated. In our previous study, an essential function was identified for cell cycle progression for the RNA binding protein RBM8A in A549 cells (19). Knockdown of the *RBM8A* gene resulted in arrest at the G_2_/M phase, concomitant with aberrant centrosome formation. In addition, these cells underwent apoptosis following *RBM8A* knockdown. On the other hand, in our recent study, immunostaining experiments showed that RBM8A proteins were localized at centrosomes and microtubules (20). This was confirmed by the presence of exogenous tagged RBM8A in A549 cells. These results prompted the study of the localization of RBM8A proteins with respect to paracrystals in the present study.

Recent progress in using the exogenous expression of fluorescent proteins that are conjugated with polypeptides via baculovirus infection has enabled simple, rapid visualization of target proteins in living cells (21–23). This can be combined with time-lapse microscopy and can be used to make movies of living cells (24,25). The present study aimed to develop vinblastine-induced paracrystalline aggregate formation in a human lung tumor cell line and establish a time-lapse analysis system.

## Materials and methods

### Cell culture and introduction of labeled proteins

The human non-small cell lung cancer A549 cell line (Riken Tsukuba Institute, Tsukuba, Japan) was maintained in Dulbecco’s modified Eagle’s medium (Sigma-Aldrich, St. Louis, MO, USA), supplemented with 10% fetal bovine serum (Sigma-Aldrich) and antibiotics [penicillin (100 units/ml) and streptomycin (100 units/ml) solution; Wako Pure Chemicals Co., Ltd., Osaka, Japan]. A total of 40,000 cells were seeded onto a glass-bottomed dish (Asahi Glass Co., Ltd., Tokyo, Japan). The cells were allowed to adhere and grow for two days at 37°C in 5% CO_2_ prior to applying Cellular Lights™ (Invitrogen Life Technologies, Carlsbad, CA, USA) transduction.

### Introducing Cellular Lights

To introduce fluorescent proteins conjugated with proteins, Cellular Lights Red Fluorescent Protein (RFP)-Tubulin and Cellular Lights Green Fluorescent Protein (GFP)-Actin were introduced at the same time. Cellular Lights Null (empty control) was used as a negative control. All of these regents were purchased from Invitrogen Life Technologies. The reagents contained a baculovirus that enables the expression of autofluorescent proteins upon entry into insect cells. The use of baculovirus to deliver genes into mammalian cells, referred to as BacMam technology, was developed and became commercially available fairly recently (21,22). BacMam technology has the following significant features: i) High transduction efficiency, ii) minimal cytotoxic effects, iii) high expression levels, iv) safety, as it cannot replicate in mammalian cells, and v) easy delivery of multiple different genes. Thus, BacMam technology is a method of gene delivery with few or no observable side-effects. The reagents used combine fluorescent protein-tagged target proteins with the viral delivery used with BacMam technology, which results in extensive expression in mammalian cells. All reagents were used according to the manufacturer’s protocol.

In brief, the BacMam enhancer solution was prepared by reconstituting an entire vial of enhancer in dimethyl sulfoxide (Sigma-Aldrich). The transduction solution was prepared by combining a Cellular Lights reagent with Dulbecco’s phosphate-buffered saline (D-PBS, Wako Pure Chemicals Co., Ltd.). To simultaneously transduce cells with RFP-Tubulin and GFP-Actin, the additional volume of the reagents was substituted for PBS. Subsequent to aspirating the transduction solution from the cell culture dish, culture medium with or without serum plus enhancer was added. The cells were subsequently incubated for 2 h. Furthermore, the cells were incubated at 37°C in 5% CO_2_ for 16 h, during which time the introduced fluorescent proteins were expressed.

### Expression of cell components and time-lapse analysis

Time-lapse imaging was performed using an incubator microscope system (LCV110; Olympus Corp., Tokyo, Japan). Vinblastine sulfate (30 μM; Calbiochem, La Jolla, CA, USA) was added to the cells in which the Cellular Lights reagents had been introduced. The glass-based dishes were set up in a tray for the LCV110 system and preincubated for 60 min. Image acquisition was begun using a time interval of 15–20 min between each acquisition. These images were processed using the MetaMorph software (Molecular Devices, Inc., Sunnyvale, CA, USA).

### Immunostaining

The cells were fixed in 4% paraformaldehyde, followed by treatment with 0.2% Triton X-100 (Sigma-Aldrich). Next, either a polyclonal primary goat antibody against tubulin (1:200; Santa Cruz Biotechnology, Inc., Santa Cruz, CA, USA) or a mouse anti-human RBM8A monoclonal antibody (1:1,000; clone 4C4, Sigma-Aldrich) was incubated with the fixed cells. Alexa Fluor 488 (green) or 589 (red) conjugated secondary antibodies (Molecular Probes; Invitrogen Life Technologies) were used as appropriate. Cell nuclei were stained with DAPI. Prolong Gold anti-fade reagent (Invitrogen Life Technologies) was used to avoid fading. Images were acquired using an Axiovert 200M microscope (Carl Zeiss, Oberkochen, Germany) and processed using ZEN software (Carl Zeiss).

## Results

### Vinblastine-induced paracrystal formation in A549 cells

**Figure 1:**
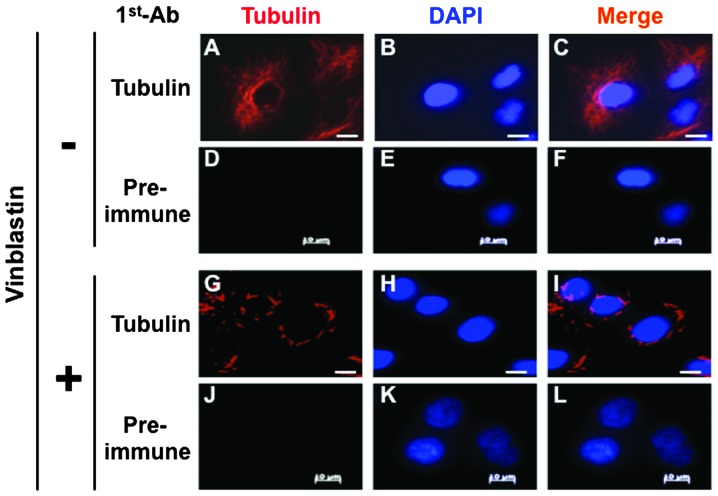
Paracrystal formation in human A549 cells. A549 cells were treated with a vinblastine solution and fixed. The cells were stained with an anti-tubulin antibody followed by an appropriate secondary antibody. Nuclei were stained with DAPI. Images were acquired using an Axiovert 200M microscope. Bar = 10 μm.

To confirm paracrystal formation in the A549 cells, the cells were treated with vinblastine for 12 h and the paracrystals that formed were stained with an anti-tubulin antibody, as described in previous studies (26,27). As shown in Fig. 1A–C, tubulin cytoplasmic localization was verified in the cells without vinblastine treatment. In the vinblastine-treated cells, characteristic paracrystal tube-like structure formation could clearly be detected (Fig. 1G–I). Thus, vinblastine treatment caused paracrystal formation in the A549 cells.

### RFP-Tubulin co-localizes with tubulin staining

**Figure 2:**
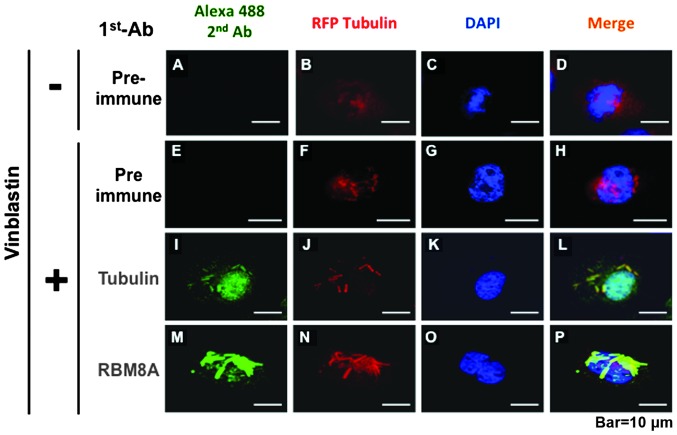
Overlapping RFP-Tubulin immunostaining. RFP-Tubulin was introduced into the A549 cells, which were immunostained with either an anti-tubulin antibody or an anti-RBM8A antibody and appropriate secondary antibodies conjugated with Alexa 488. Images were acquired using an Axiovert 200M microscope. Bar = 10 μm.

RFP-Tubulin was introduced and immunostaining was performed with an anti-tubulin antibody. As shown in Fig. 2, the RFP-Tubulin was expressed in the A549 cells and was localized to the centrosomes (Fig. 2A–D). The signals from immunostaining with an anti-tubulin antibody co-localized with those of RFP-Tubulin. Thus, RFP-Tubulin could be used to detect the formation of paracrystals, which were identified by an anti-tubulin antibody (Fig. 2I–L).

At the same time, a mouse anti-RBM8A antibody (Sigma-Aldrich) was used, as our previous study had indicated that this protein localized to centrosomes. Our preliminary results showed vivid staining of the spindle fibers, in addition to the centrosomes, when using this antibody (20). Thus, we speculated that the anti-RBM8A antibody from Sigma-Aldrich could co-localize with paracrystals. As shown in Fig. 2M–P, the signals from immunostaining with the anti-RBM8A antibody overlapped with the RFP-Tubulin signals. Thus, this monoclonal antibody was useful for the detection of paracrystals. In addition, in place of the antibody from Sigma-Aldrich, a self-made rabbit antiserum against the N-terminal region of RBM8A was used and similar results were obtained (data not shown). Thus, it was concluded that RNA-binding RBM8A proteins that were localized to paracrystals formed due to vinblastine treatment.

### Observations of vinblastine-induced paracrystals by Cellular Lights

**Figure 3:**
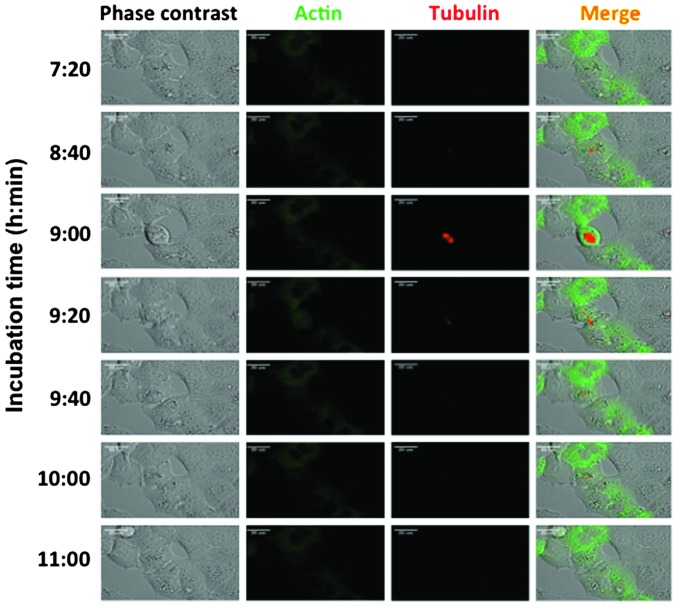
Observations of fluorescently-labeled tubulin and actin. Red fluorescent protein-tubulin and green fluorescent protein-actin were introduced into the A549 cells. The cells were observed using an LCV110 system. Representative images of control A549 cells are shown. The duration of incubation is presented by the horizontal axis (magnification, ×250).

To obtain time-lapse images of paracrystal formation, we introduced Cellular Lights RFP-Tubulin into the A549 cells and observed the cells using an Olympus LCV110 system. To determine the outline of each cell, GFP-Actin was also introduced. Fig. 3 shows the results of the time-lapse analysis of the cells without vinblastine treatment using the LCV110 system and the progression of the mitotic phases. Green signals were derived from GFP-Actin and red signals were derived from RFP-Tubulin. Microtubule formation during mitotic phases was observed by RFP-Tubulin at the 9 h 00 min time-point, and GFP-Actin was spread through the entire cytoplasm.

**Figure 4:**
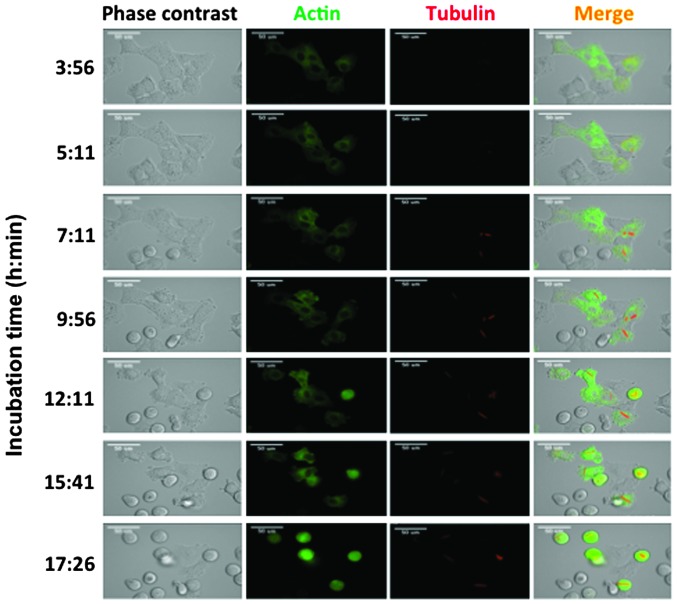
Time-lapse observations of paracrystal formation in viblastine-treated A549 cells. Cells with fluorescently-labeled tubulin and actin were observed using an LCV110 system. Incubation times are shown on the left of the images. Duration of incubation is presented by the horizontal axis (magnification, ×140).

In comparison, the time-lapse results for the vinblastine-treated cells are shown in Fig. 4 (images obtained on different days). Tube-like structures were clearly demonstrated by the red signals derived from RFP at the 7 h 11 min time-point. These paracrystals gradually grew until the 12 h 11 min time-point. During paracrystal formation, cell cycle progression was arrested around mitosis, as the cell shapes were round. At the same time, the green signals derived from GFP spread as shown in the negative control cells. Subsequent to the 15 h 41 min time-point, rounded cells predominantly appeared and cell cycle progression was inhibited. These cells retained paracrystals and appeared to be arrested at the mitotic phase.

**Figure 5:**
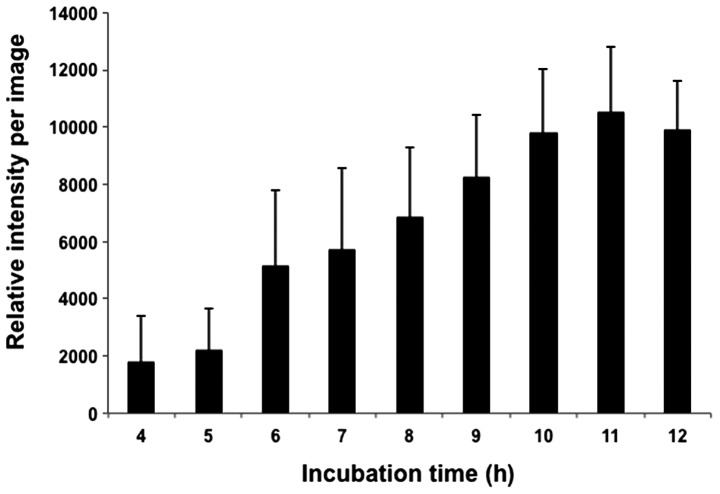
Red fluorescent protein (RFP)-derived fluorescence intensity. RFP intensities per image were determined in five randomly selected independent images and the mean and standard deviation were calculated.

Finally, paracrystalline RFP fluorescence intensity was measured on these time-lapse images, which showed the time dependency for their formation (Fig. 5). Based on the images in Fig. 4, RFP fluorescence intensity gradually increased and the green signals were stably expressed.

## Discussion

The immunostaining results of the present study revealed overlapping signals from an anti-tubulin antibody and an anti-RBM8A antibody in A549 cells. In a previous study, localization of syndecan proteins to tubulin was reported on the basis of its localization to vinblastine-induced paracrystals (13). Similar to this study, the co-localization of RBM8A to paracrystals in the present study supports our previous observations that RBM8A localizes to microtubules and centrosomes (20). As mRNA-protein complexes are known to move via their binding to microtubules (28), RBM8A localization may indicate that certain mRNA molecules on microtubules bind to RBM8A. To examine whether RBM8A is required for paracrystalline formation, we repeatedly performed knockdown experiments in A549 cells, but these trials resulted in rapid cell death and did not obtain clear results (data not shown).

In the present study, a time-lapse analysis system was successfully established. The combination of time-lapse analysis with RFP-tubulin expression is the main technique analyzed in the present study. This method was anticipated to obtain more detailed information compared with conventional immunostaining methods with fixed cells. Staining became visible following spindle fiber or paracrystal formation. RFP-Tubulin was expressed by Cellular Lights and the molecules spread through the cytoplasm. RFP-tubulin was not evident as the proteins were diffuse. When RFP-tubulin forms microtubules or paracrystals due to polymerization, it becomes clearly visible.

As immunostaining with an RBM8A antibody successfully detected paracrystals in these cells, the expression of GFP-tagged RBM8A proteins was attempted in the A549 cells. The fluorescent protein-tagged RBM8A also appeared to be useful for the detection of paracrystals, similar to RFP-tubulin in the present study. However, transient expression of this modified RBM8A protein caused rapid cell death similar to that observed in the knockdown experiments (data not shown) and it was therefore concluded that this was not suitable for detection. In the case of time-lapse analysis, in future studies, combinations with other cell cycle progression markers, such as a fluorescent ubiquitination-based cell cycle indicator system (29,30), will enable the analysis of cell cycle-dependent mechanisms.
